# Using a Device-Free Wi-Fi Sensing System to Assess Daily Activities and Mobility in Low-Income Older Adults: Protocol for a Feasibility Study

**DOI:** 10.2196/53447

**Published:** 2024-11-12

**Authors:** Jane Chung, Ingrid Pretzer-Aboff, Pamela Parsons, Katherine Falls, Eyuphan Bulut

**Affiliations:** 1 Nell Hodgson Woodruff School of Nursing Emory University Atlanta, GA United States; 2 School of Nursing Virginia Commonwealth University Richmond, VA United States; 3 College of Engineering Virginia Commonwealth University Richmond, VA United States

**Keywords:** Wi-Fi sensing, dementia, mild cognitive impairment, older adults, health disparities, in-home activities, mobility, machine learning

## Abstract

**Background:**

Older adults belonging to racial or ethnic minorities with low socioeconomic status are at an elevated risk of developing dementia, but resources for assessing functional decline and detecting cognitive impairment are limited. Cognitive impairment affects the ability to perform daily activities and mobility behaviors. Traditional assessment methods have drawbacks, so smart home technologies (SmHT) have emerged to offer objective, high-frequency, and remote monitoring. However, these technologies usually rely on motion sensors that cannot identify specific activity types. This group often lacks access to these technologies due to limited resources and technology experience. There is a need to develop new sensing technology that is discreet, affordable, and requires minimal user engagement to characterize and quantify various in-home activities. Furthermore, it is essential to explore the feasibility of developing machine learning (ML) algorithms for SmHT through collaborations between clinical researchers and engineers and involving minority, low-income older adults for novel sensor development.

**Objective:**

This study aims to examine the feasibility of developing a novel channel state information–based device-free, low-cost Wi-Fi sensing system, and associated ML algorithms for localizing and recognizing different patterns of in-home activities and mobility in residents of low-income senior housing with and without mild cognitive impairment.

**Methods:**

This feasibility study was conducted in collaboration with a wellness care group, which serves the healthy aging needs of low-income housing residents. Prior to this feasibility study, we conducted a pilot study to collect channel state information data from several activity scenarios (eg, sitting, walking, and preparing meals) using the proposed Wi-Fi sensing system continuously over a week in apartments of low-income housing residents. These activities were videotaped to generate ground truth annotations to test the accuracy of the ML algorithms derived from the proposed system. Using qualitative individual interviews, we explored the acceptability of the Wi-Fi sensing system and implementation barriers in the low-income housing setting. We use the same study protocol for the proposed feasibility study.

**Results:**

The Wi-Fi sensing system deployment began in November 2022, with participant recruitment starting in July 2023. Preliminary results will be available in the summer of 2025. Preliminary results are focused on the feasibility of developing ML models for Wi-Fi sensing–based activity and mobility assessment, community-based recruitment and data collection, ground truth, and older adults’ Wi-Fi sensing technology acceptance.

**Conclusions:**

This feasibility study can make a contribution to SmHT science and ML capabilities for early detection of cognitive decline among socially vulnerable older adults. Currently, sensing devices are not readily available to this population due to cost and information barriers. Our sensing device has the potential to identify individuals at risk for cognitive decline by assessing their level of physical function by tracking their in-home activities and mobility behaviors, at a low cost.

**International Registered Report Identifier (IRRID):**

DERR1-10.2196/53447

## Introduction

### Background

Mild cognitive impairment (MCI) is underdetected in the community. About 15% of individuals with MCI progress toward Alzheimer disease (AD)/AD-related dementias (ADRD) after 2 years [1]. Older adults with health disparities, defined here as those with low socioeconomic status and racial minorities, are at increased risk of developing AD/ADRD due to poorly managed health conditions, structural racism, and barriers to health care access [2-4]. The United States federal poverty level definition of low income for a single-person household is US $15,060 in 2024. Resources to assess functional deterioration and detect the risk of progression to AD/ADRD are further limited in this population. Research suggests that functional decline begins years before clinical symptoms of AD/ADRD [5]. Therefore, innovative, accessible, and easy-to-use approaches are imperative to capture these functional changes early, intervene before symptoms arise, and provide quality care [6].

Older adults with MCI are at greater risk for physical function disability, such as limitations in activities of daily living (ADLs), instrumental activities of daily living (IADLs), and mobility behaviors [7-10]. For example, cognitive impairment affects the ability to perform and complete daily activities such as meal preparation and using electronic devices (eg, microwaves, computers, and telephones). Individuals with MCI are also more likely to be less mobile and engage in sedentary behaviors [8,11,12]. Limitations in daily activities threaten older adults’ quality of life and well-being [13]. It is essential to monitor daily activities and mobility to capture early cognitive decline for a timely intervention. Failure to recognize early impairment in a timely manner is likely to lead to disability and adverse health outcomes such as falls, institutionalization, decreased quality of life, and mortality.

With the emergence of novel smart home technologies (SmHT) and advanced data analytics, it is possible to sense motion, presence, and other behavioral indicators continuously and with less intrusion into the daily lives of older adults. SmHT provides an objective assessment of activity and mobility that is not subject to the effects of recall bias or memory impairment [14-16]. Although these solutions have effectively identified digital health indicators, such as gait speed, variability over time [17], or sleep disturbance [18], these sensors cannot label specific activity types. The ability to identify and differentiate human activities will be improved when algorithms to disambiguate the data are fully available. Wearable sensors have been proposed to obtain objective, high-frequency, and remote monitoring [19-21]. The data collected from these sensors are fed into complex machine learning (ML) models (eg, long short-term memory and Gaussian Naive Bayes classifier) to recognize ADLs and identify anomalies [22-24]. However, these devices are not easy to use by those with MCI [25]. Moreover, these devices typically necessitate the use of a smartphone app for data syncing. However, it is worth noting that a significant portion of older adults facing health disparities may not possess smartphones, lack proficiency in using smartphone apps even if they own them, or have limited IT literacy [26,27].

Alternatively, Wi-Fi signal–based sensing solutions could mitigate this issue. We propose an ML-based data processing method for extracting discriminative activity features from Wi-Fi signals. Human activities incur changes in the signal received by a nearby Wi-Fi device [28], resulting in distinct patterns of wireless signals surrounding us. There are several advantages of using Wi-Fi signal–based sensing. First, it enables device-free wireless localization and activity recognition [29]. It is possible to sense the location and activity of an individual without equipping them with any wearable device. Second, this sensing technology allows for more accurate capturing of daily activities and mobility, even in situations with poor lighting or obstructed camera views, due to its capability to penetrate walls or furniture. Third, compared to the classic methods for tracking individuals and their activities (eg, global positioning system, inertial measurement unit, or video surveillance systems), this technology is lower-cost, less intrusive, and simpler to deploy [28,29]. Therefore, it holds promise for application in scalable methods for monitoring the types and levels of daily activities and mobility among older adults.

Wi-Fi sensing technology has been tested in the context of activity and gesture recognition, object sensing, and localization [30-34]. Using these Wi-Fi-sensing–based capabilities, researchers started to develop methods to apply Wi-Fi sensing to health care such as motion recognition and localization, fall detection, smoking behavior monitoring, and vital signs monitoring (eg, heart rates and breathing) [35]. Despite the potential of this technology as a low-cost in-home monitoring tool, this technology has primarily undergone deployment and assessment within controlled laboratory environments, often involving activities carried out by volunteers following specific prompts, and often within relatively brief timeframes [36]. To our knowledge, no study exists that tests its use for everyday functioning assessment in a natural setting with older adults over time. Review studies indicate that there is room for improvement in Wi-Fi health care sensing such as multisubject sensing, implementation of the setup, fine-grained motion sensing, and the need for massive datasets for training for deep learning model development [30,35]. Therefore, our goal is to develop and evaluate an innovative, affordable Wi-Fi sensing–based, nonintrusive system for tracking activity and mobility in a real-life setting involving older adult residents of low-income senior housing. This will be achieved through a week-long study in which each individual will engage in activities as per their daily routine. This research also shows the feasibility of using a collaborative transdisciplinary team approach to integrate engineering into behavioral science and ADRD research to provide tools to address MCI risk assessment challenges among older individuals with limited resources.

This proposed research aims to tackle unmet needs and address brain health challenges affecting older adults with health disparities. This research leverages our team’s multidisciplinary skillsets related to sensor-based mobility assessment (nursing, JC [14,37]; nursing, IP-A [38,39]), Wi-Fi sensing signal processing and ML/artificial intelligence (engineering, EB [34,40,41]), technology acceptance (nursing, JC [42,43]), and health and wellness in older adult residents of low-income housing (nursing, PP [44,45]; nursing KF [45,46]). The use of a nurse-driven method to guide ML training for Wi-Fi sensing technology-based assessment could help address the gap that exists regarding transferring clinical knowledge from nursing to engineering in SmHT solution development [47]. This nurse-driven method will be useful for generating clinically relevant ground truth that is associated with real features of older adults’ functional health. The outcomes of this study will offer valuable insights into the feasibility of implementing this innovative, cost-effective sensing solution to gather significant data on in-home activities and mobility.

### Aims

This is a feasibility study to develop a novel channel state information (CSI)–based device-free, low-cost Wi-Fi sensing system and associated algorithms for localizing and recognizing different patterns of in-home activities and mobility in older adult residents of low-income housing with and without MCI. The proposed study aims to (1) test the feasibility of developing a novel CSI-based device-free Wi-Fi sensing system using ML classification to identify in-home daily activities and mobility, and (2) explore the acceptability of the Wi-Fi sensing system and implementation barriers in the low-income housing setting.

## Methods

### Study Design

This is a feasibility testing study to develop and implement a low-cost Wi-Fi sensing–based nonintrusive activity and mobility assessment system.

### Ethical Considerations

Institutional review board approval has been granted on January 30, 2023 (Virginia Commonwealth University; HM#20024269). Potential participants are required to provide written informed consent to install the Wi-Fi sensing technology and other related devices in the home. Participants are informed that they have the ability to opt out of the study at any time. Except for the videotaping data of in-home activities used as ground truth, all other data will be anonymized. We provide a US $50 gift card at baseline and another US $50 at the end of the sensor-based monitoring period.

### Preliminary Work

Our prior work with the Wi-Fi sensing system involved (1) experiments in a controlled setting, and (2) a pilot study in a natural setting.

**Figure 1:**
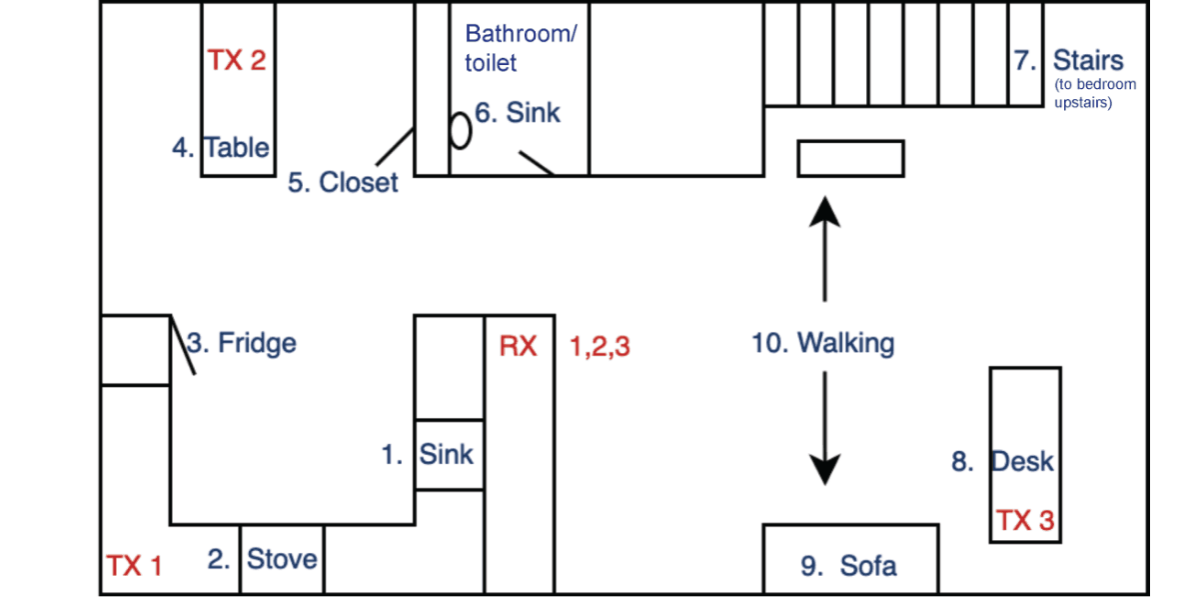
The floor map of the environment used and the activities performed during preliminary experiments. TX: Transmitter; RX Receiver (see Table 1 to see labels for activities #1-10).

First, the team conducted a series of preliminary experiments in a controlled setting. We asked a volunteer to engage in 10 distinct activities within an apartment (Table 1), with the floor plan shown below (Figure 1). The individual completed each of these activities once throughout the course and repeated this course 20 times. We used three Transmitter (TX)-Receiver (RX) pairs as marked in Figure 1. The devices were positioned to have each activity be performed close to the line-of-sight of at least one TX-RX pair so that good sensing accuracy could be achieved. For example, kitchen activities (Activity ID #1-3 in Figure 1) are best monitored by TX1-RX1 pair, while dining room and bathroom activities (#4-6) and living room activities (#7-10) can be best sensed by TX2-RX2 and TX3-RX3 pairs, respectively. We collected CSI data from each of these activities and developed simple deep learning models for detecting these activities through classification. We used data from the first 10 courses for training the ML model and the remaining 10-course data for testing the model.

**Table 1:** Preliminary accuracy of ML^a^ model for detecting 10 in-home activities.

Activity type and number		Preliminary accuracy (%)
**Kitchen activities**		93.8 (using TX1-RX1)^b^
	#1: Washing dishes at the sink	96.7
	#2: Cooking on stove top	00.4
	#3: Opening and closing the refrigerator door	93.5
**Dining room and bathroom activities**		100 (using TX2-RX2)
	#4: Writing in a notebook at the dining table	100
	#5: Opening and closing closet door	100
	#6: Washing hands in a bathroom	100
**Living room activities**		80.8 (using TX3-RX3)
	#7: Walking up and down stairs	86.2
	#8: Writing on the desk	67.5
	#9: Sitting on the sofa	83.8
	#10: Walking around	77.3

^a^ML: machine learning.

^b^TX: transmitter and RX: receiver.

Table 1 shows the preliminary accuracy values of ML model–based in-home activity detection. Our results showed that the model identified the kitchen activities with 93.8% accuracy, dining room and bathroom activities with 100% accuracy, and living room activities with 80.8% accuracy. We found that the lower accuracy in identifying living room activities was mostly due to a confusion of classification between activity #8 and activity #9. These activities shared similarities, such as limited body movements, and their locations were relatively close to each other. It is anticipated that this confusion can be resolved by gathering more data from these activities and strategically placing additional TX-RX pairs closer to their locations. Even though the model was based on a limited set of data, these preliminary results show that the proposed system is promising.

**Figure 2:**
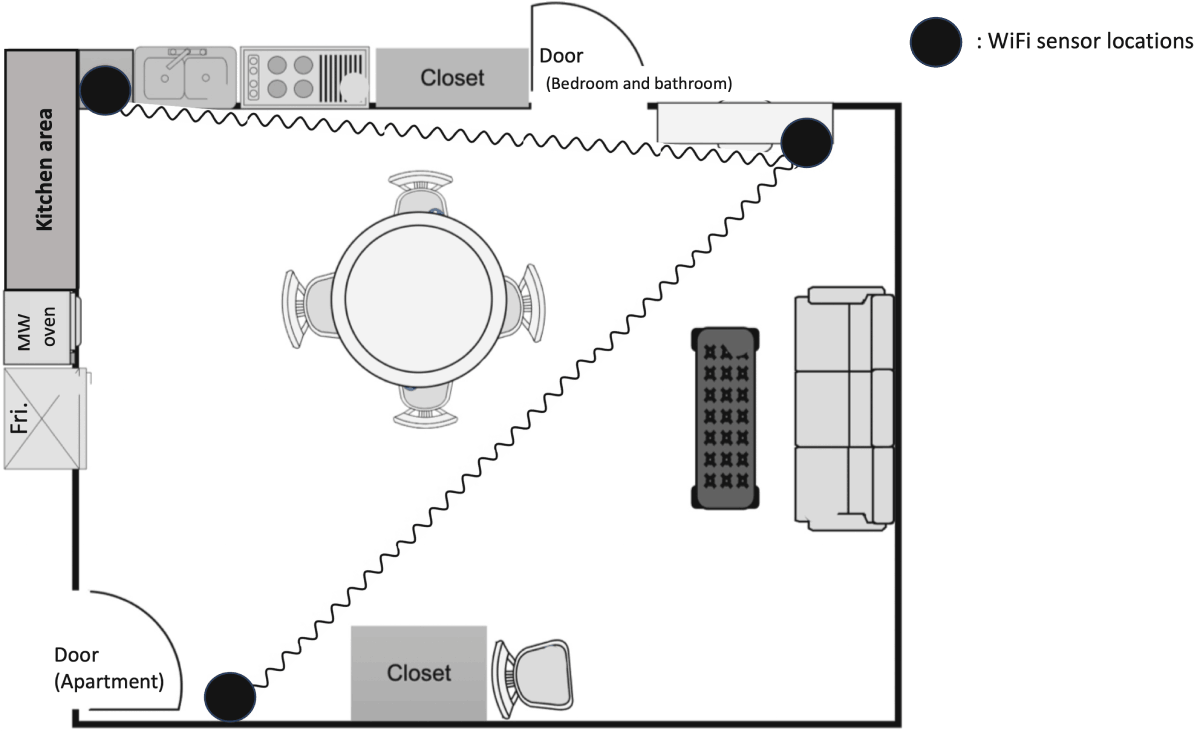
A participant’s floor map of a one-bedroom apartment with the Wi-Fi sensor setup.

In the second phase, we conducted a pilot study with two older adult residents of low-income housing. The sensing system was deployed for about 5-7 days in each residence and tracked in-home activities (eg, cooking, eating, and opening a refrigerator) and mobility behaviors (eg, entering the apartment and entering a bedroom). Figure 2 shows one participant’s apartment floor plan with the Wi-Fi sensor setup.

**Figure 3:**
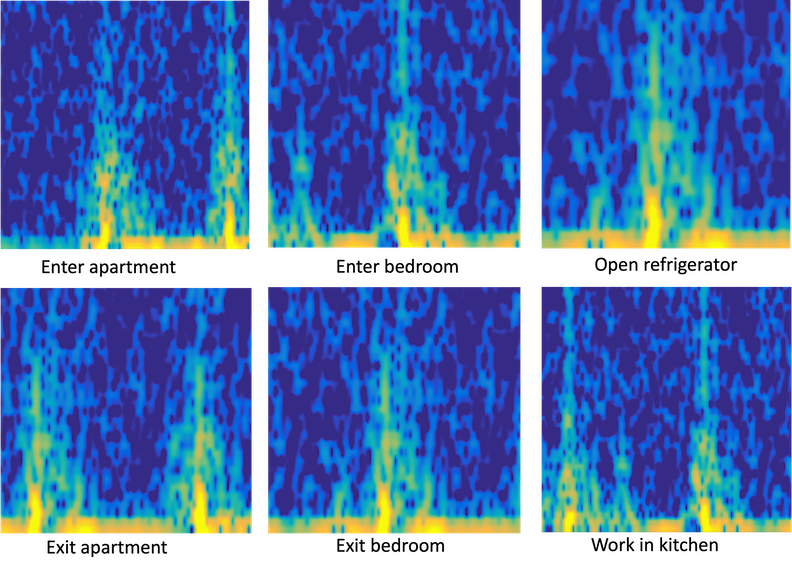
Spectrogram images obtained from six different activities of the first participant.

This pilot study demonstrated the successful deployment of the Wi-Fi sensing system in real-world environments of older adults, recruitment feasibility, and acceptance of the technology among the participants [48]. We developed an initial ML model to establish the accuracy of activity detection using preprocessed raw data and video-based ground truth. The model, based on the data from this participant, achieved 68.8% accuracy in recognizing six different activities, such as entering an apartment, entering a bedroom, opening a refrigerator, exiting an apartment, exiting a bedroom, and kitchen activities. Figure 3 shows the spectrogram images obtained from these activities.

To ensure participant privacy throughout the pilot study, we implemented several measures. First, participants were instructed to wear clothing while in the monitored areas, such as the living room and kitchen, minimizing any potential for privacy breaches. Second, during the informed consent process, we ensured that participants would not bring any other visitors during the monitoring period. Additionally, during the annotation process of activities on video recordings, any footage depicting naked bodies was promptly identified and removed. These steps were taken proactively to maintain the privacy of our participants, particularly considering the sensitive nature of activities that may occur in areas, such as the bathroom and bedroom, which were deliberately excluded from sensor and camera deployment.

### Participants and Recruitment

For this study, the sensing system will be installed over a week in the apartments of 10 older adults with MCI and 10 older adults without MCI. The age criterion for defining older adults varies, but adults 65 years and older are typically considered older adults by the US Census Bureau and Medicare. However, we decided to choose the sample with an age criterion older than 60 years to include a broader pool of individuals with varying levels of physical and cognitive function given that cognitive impairment can begin before age 60 [49]. Textbox 1 lists inclusion and exclusion criteria.

**Inclusion and exclusion criteria.**

**Inclusion criteria**

- Age ≥60 years
- Living alone in low-income senior housing in the greater Richmond area, Virginia, that is subsidized by the US Department of Housing and Urban and other sectors
- Non–mild cognitive impairment (non-MCI) group, determined by the Montreal Cognitive Assessment (MoCA) score≥24; MCI group (n=10), determined by the MoCA score<24 and≥18

**Exclusion criteria**

- Severe visual or hearing impairment
- Severe motor disturbances (eg, wheelchair-bound and neurodegenerative diseases)
- Having a pet
- Inability to speak and understand English

The Mobile Health and Wellness Program (MHWP, previously known as the Richmond Health and Wellness Program) at Virginia Commonwealth University School of Nursing is the source of collaboration and research subjects for the proposed project [2]. MHWP is an interprofessional wellness care program for independent-living seniors at low-income housing facilities. MHWP coordinates with local services to provide health education and assessments at 5 low-income housing facilities in Richmond, Virginia, United States. We will recruit participants from these sites. Recruitment will be conducted via multiple mechanisms using institutional review board–approved materials, site information sessions, direct referrals from the MHWP staff, and study flyers.

### Data Collection Procedures

#### Initial Contact and Screening Process

If interested, older adults will be encouraged to contact the study coordinator via telephone or in person. During the initial contact, the potential participants will be provided an overview of the study and screened to ensure they meet eligibility criteria. Further details about the study will be explained, and the study staff will answer any questions that participants have at that time. At all times, we will emphasize the voluntary nature of participation and the ability to withdraw without consequence.

#### Baseline Data Collection (Visit 1)

Following informed consent, we will administer questionnaires to assess participants’ demographics, physical function, and psychosocial health. We will then ask the participants for a set of daily activities they usually perform in their living spaces such as where they sit (eg, couch and dining table), eat, lie down, prepare meals, use the computer or tablet (if any), and how much time they usually spend in each room and out of the home.

#### Wi-Fi Sensing System Installation (Visit 2)

**Figure 4:**
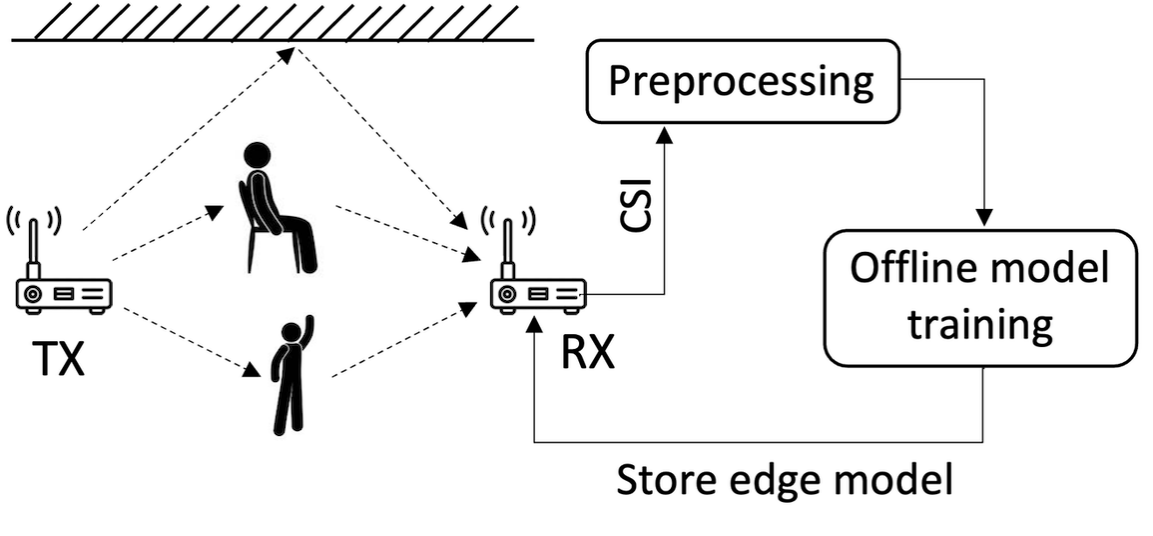
Overview of a typical Wi-Fi sensing system. CSI: channel state information.

Depending on the floor plan and the locations of the activities to be sensed, we will deploy the sensing system using as many TX-RX pair devices as needed to ensure each activity is in the line-of-sight of at least one TX-RX pair. Figure 4 shows how a typical Wi-Fi sensing system works. The sensing system will be left for a week at home to collect data for evaluating the system. To obtain ground truth values, we will place video cameras in several locations in the apartment (except the bathroom and bedroom to protect privacy) to monitor all tracked activities accurately. Once the evaluation period ends, we will use these recordings to label activities in the CSI data based on their timing.

#### Sensor Removal and Exit Interviews (Visit 3)

Approximately one week after the sensor-based monitoring starts, the sensing system and video cameras will be removed. The collected data will be used to evaluate the system’s accuracy by comparing the Wi-Fi sensing data with ground truth values. During this visit, we will conduct an individual qualitative interview to explore their perceptions and attitudes toward the sensing system. Specifically, informed by the Unified Theory of Acceptance and Use of Technology [50], we will assess perceived usefulness and behavioral intention to adopt a Wi-Fi sensing system [51]. The interviews will also provide feasibility outcomes, such as implementation barriers (eg, privacy and security concerns) and facilitators (eg, health monitoring needs, peer influence, and cultural values), and recommended changes for future system deployment. Textbox 2 shows semistructured qualitative interview questions we developed.

**Semistructured qualitative interview script.**

- Please tell me two things you liked and two things you wondered (or “worried”) about the sensor devices (cues: comfortable or uncomfortable, nervous, worried, and confused).
- Thinking back to the time when you heard about this study, what made you decide to participate in this study? Did you have any concerns about privacy, being monitored, or your information being breached and shared without your agreement?
- In general, what concerns did you have about the sensor devices? (cues: being monitored, inappropriate sharing of data, maintenance issues, etc)
  Did you have any privacy concerns about the video cameras? If so, would you explain further?
  (If the participant mentioned an abstract term, such as “privacy,” “confidentiality”): Could you tell me specifically what types of things or examples you were thinking about when you said “privacy”?
- How often did you think about these devices? Do you think these devices have changed the way you carry out your daily activities? (cues: behaving differently, leaving out more frequently, doing more exercise, etc) If so, in what way? What activities have changed?
- How would the people around you react if you used these devices at home or if they saw them?
  Why do you think they would react that way?
- Would you like to be able to turn the sensor system on and off depending on your preferences at any given time?
- This sensor system generates data about the timing, duration, and frequency of your daily activities and movement and shows how active you are in your home. For example, cooking, washing dishes, eating, sitting on the couch, sitting at the desk, walking, etc. Would this type of data have any usefulness for you?
- Would you like to see the data from the sensor system about your activity?
  Would you be willing to share this data with your family? Would you explain why or why not?
  How about friends and health care providers? Why and why not?
- Would you like to have this system if it were available in the future? Why or why not?
- We’ve already touched on privacy issues. Can you think of any other issues you ran into while you were living with these devices?
- Are there any other thoughts you would like to share about this technology or this research study?

### Data Processing and Analysis

#### Wi-Fi Sensing–Based ML Model Development

The collected CSI data and its labels will be used to develop an ML model. For the ML model, we will initially consider a lightweight dense neural network classifier architecture in order to fit the model in the low-memory ESP32 devices. However, for performance comparison, we will also consider developing more complex ML models (eg, convolutional neural networks, recurrent neural networks, and long short-term memory) with a different number of hidden layers. From the ML model, several Wi-Fi sensing–based metrics will be extracted such as the daily number and duration of each of the selected in-home activities and mobility behaviors.

Table 2 shows examples of activity features that will be captured by Wi-Fi sensing technology. These activities are categorized into IADL, ADL, or mobility. The majority of activities listed under the daily activities category in Table 2 relate to IADL. For instance, “Sitting in the dining table” may indicate engagement in “writing” or “technology device use” within the IADL context, or it may signify “eating” within the ADL context. Activities listed under the mobility behaviors category are specifically categorized as life-space mobility-related actions.

**Table 2:** Examples of anticipated activity and mobility features to extract and analyze from Wi-Fi sensing data.

Category and activity type		Definition
**Daily activities**		
	Fridge time	The first moment the person touches the door to open it and actively performs fridge activity (eg, shuffling items around in the freezer)
	Sink use	The start and end time of standing at the sink or faucet use time
	Sitting in the dining area	The start and end time the body touches the dining chair and gets off the chair
	Cooking	The start time and end time of stove use or microwave use
	Phone use	The time the person starts using a phone or tablet (eg, text, app use, and phone call) and the time the person stops
**Mobility behaviors**		
	Entering apartment	The time the person opens the front door and to the end time the person closes the door to get into the apartment
	Exiting apartment	The time the person opens the front door and the time the person closes the door to exit
	Entering a private area (eg, bedroom or bathroom)	The time the person passes a bedroom or bathroom door or threshold to enter a private area
	Exiting a private area	The time the person passes a bedroom or bathroom door or threshold to exit from a private area
	Sitting on sofa	The start and end time the body touches the sofa and gets off the sofa (sitting position, does not include the lying position)
	Lying on sofa	The start time as the time the participant lies horizontally and end time as the time the participant returns to a sitting position
**Other**		
	Second person	The time the person appears and disappears from space

#### Qualitative Interviews

Interview data will be analyzed using thematic analysis [51] and inductive coding method [52] to obtain insights into user perceptions, needs, barriers, and concerns that could influence the sensing system adoption and implementation in the future, especially in the low-income community setting. Audio files will be transcribed verbatim by a professional transcriptionist. Transcripts will be coded line-by-line by the analysis team (JC and IP-A). The team will conduct the initial analysis independently identifying significant statements from transcripts to develop codes. The codebook will then be developed collaboratively through regular meetings. Once the codebook is refined, the transcripts will be analyzed independently again by the coding team. The team will then meet to reach a consensus on the coding structure and develop related thematic categories. Data will be grouped and reviewed for trends, patterns, and ideas that provide insight into older adults’ attitudes and perceptions of the sensor system. The analysis will be organized into an expanding list of themes arising from content, concepts, and descriptive categories. In order to ensure credibility, transferability, dependability, and confirmability, we will keep field notes, reflexive journaling, and audit trail and have team debriefings to discuss our reflections [53].

Table 3 shows specific methods for achieving each study aim.

**Table 3:** Study aims and specific methods and their focus to achieve each aim.

Aim and method		Focus
**Aim 1: to test the feasibility of developing a novel CSI^a^-based device-free Wi-Fi sensing system using ML^b^ classification to identify in-home daily activities and mobility**		
	Ground truth data annotations ML model development	Evaluation of the accuracy of Wi-Fi sensing–based ML models in activity detection
	Screening and enrollment log analysis	Recruitment feasibility
**Aim 2: To explore the acceptability of the Wi-Fi sensing system and implementation barriers in the low-income housing setting**		
	Qualitative interviews	Wi-Fi sensing technology acceptance, privacy concerns, and factors leading to technology implementation

^a^CSI: channel state information.

^b^ML: machine learning.

## Results

This project was funded in June 2023. Participant recruitment began in July 2023. We screened 10 individuals and obtained informed consent from 6 older adults. Four others either changed their minds before or during the consenting process. Among the 6 individuals who consented, 5 individuals were enrolled in the study and completed all the study activities. Recruitment and data collection is ongoing. We expect preliminary results to be available in the summer of 2025.

## Discussion

### Study Significance and Future Research

This study will investigate the feasibility, accuracy, and acceptability of the new sensing system in community-dwelling older adults with health disparities that can characterize and quantify daily activities in a real-world setting while also being discreet, affordable, and requiring minimal user engagement. Crucial to the successful implementation of the Wi-Fi sensing system to support older adults, a number of initial development stages are necessary. The proposed research fills the gap by experimenting with the system in the living environments of older adults and providing insights into the technology implementation in the community. This project has the potential to address unmet needs and brain health challenges among low-income older adults with chronic illnesses, disabilities, and social determinants of health.

### Privacy Considerations for Community-Engaged Artificial Intelligence/ML Development Research

Given the historical distrust within the community, particularly among African American and low-income populations, toward academia, the health care system, and researchers, it is crucial to address privacy concerns transparently. In this study, we took deliberate steps to ensure privacy by refraining from installing sensors and video cameras in sensitive areas such as the bathroom and bedroom. This decision was made following consultations with the MHWP team to uphold privacy safeguards and facilitate the recruitment process. We aimed to respect the privacy and dignity of participants while still gathering valuable data on perceptions of novel sensing technology, acceptability, feasibility, and implementation facilitators and barriers within underrepresented communities.

### ML Model Generalizability

There is a concern about the transferability of an ML model developed based on a small number of participants. There are solutions to generalize models that can provide an initial high accuracy with datasets from new people not used for training. This includes federated learning–based training of collected data from multiple individuals involved in training data collection. In our previous work [40], we shared results based on a federated learning approach and showed that we can obtain a subject-independent ML model. Moreover, with further training of this pretrained model using a few more datasets from new participants, we can customize it to this person and get much higher accuracy.

Our long-term goal is to implement this system in independent living facilities, including low-income housing, as a safety monitoring and home assessment tool for preventing further functional decline and coordinating value-based services for health and day-to-day management. This marks a crucial initial step toward early detection of MCI and the coordination of care efforts to delay the progression to AD/ADRD in older adults facing health disparities. If successful, we envision the potential application of the Wi-Fi sensing system across a broader spectrum. The system will ultimately improve the ability to assess functioning and capture early decline in community-dwelling older adults.

### Limitations

This project is a pilot study that will be performed with a small sample of community-dwelling older adults. The demographics of the study sample are not representative of other groups of older adults. Another limitation of this study is that it uses video recordings as ground truth, which could raise privacy concerns among participants. Therefore, in the future, we will explore alternative methods for obtaining ground truth data beyond video-based approaches. This may involve using various types of sensors, such as contact sensors and force sensors, attached to objects within the environment. Additionally, Wi-Fi signals can potentially be sniffed from outside of the building by malicious actors to some degree. However, this will not release any information regarding the study participant’s activities without knowing the ML model that is trained to recognize these activities from signals. Our goal is to observe a level of accuracy from the proposed device-free sensor system that is very close to the ground truth. In case of unexpected results, we will consider collecting more training data, optimizing the locations of TX and RX devices, applying additional filtering on Wi-Fi signals, and increasing the hidden layers of the ML model.

### Conclusions

This pilot study is poised to make a valuable contribution to SmHT science and ML capabilities for early detection of cognitive decline particularly among socially vulnerable older adults, because sensing devices are not readily available to this population due to cost and information barriers. Our passive sensing device has the potential to identify individuals at risk for cognitive decline by assessing their level of physical function by tracking their in-home activities and mobility behaviors. The cost-effectiveness of the Wi-Fi sensing system will enhance its overall utility and adoption in underserved low-income communities. Our future plans involve (1) using the obtained results to evaluate the Wi-Fi sensing system across a wide range of populations and contexts, encompassing diverse socioeconomic groups, as well as individuals with varying degrees of AD/ADRD; (2) evaluating the impact of the Wi-Fi sensing system use on patient and caregiver outcomes, such as functional changes, care decision-making, and resource planning; and (3) determining the effective way of sharing the collected data with essential stakeholders (eg, older adults, family caregivers, health care providers, and social workers).

## Abbreviations

AD: Alzheimer disease
ADL: activities of daily living
ADRD: Alzheimer disease–related dementia
CSI: channel state information
IADL: instrumental activities of daily living
MCI: mild cognitive impairment
MHWP: Mobile Health and Wellness Program
ML: machine learning
SmHT: smart home technology
